# Effects of Inspiratory Muscle Training on Muscle Oxygenation during Vascular Occlusion Testing in Trained Healthy Adult Males

**DOI:** 10.3390/ijerph192416766

**Published:** 2022-12-14

**Authors:** Rodrigo Yáñez-Sepúlveda, Humberto Verdugo-Marchese, Daniel Duclos-Bastías, Marcelo Tuesta, Ildefonso Alvear-Ordenes

**Affiliations:** 1 Applied Physiology Laboratory (FISAP), Institute of Biomedicine (IBIOMED), University of León, 24071 León, Spain; 2 School of Education, Pedagogy in Physical Education, Universidad Viña del Mar, Viña del Mar 2572007, Chile; 3 Laboratory of Sports Sciences, Centro de Medicina Deportiva Sports MD, Viña del Mar 2521156, Chile; 4 School of Physical Education, Pontificia Universidad Católica de Valparaíso, Valparaíso 2374631, Chile; 5 Exercise and Rehabilitation Sciences Institute, School of Physical Therapy, Faculty of Rehabilitation Sciences, Universidad Andres Bello, Santiago 7591538, Chile

**Keywords:** near-infrared spectroscopy, pulmonary function test, respiratory muscle training

## Abstract

Inspiratory muscle training (IMT) may have an additional effect on cardiovascular autonomic modulation, which could improve the metabolism and vascular function of the muscles. Aim: To determine the effects of IMT on vascular and metabolic muscle changes and their relationship to changes in physical performance. Methods: Physically active men were randomly placed into an experimental (IMTG; *n* = 8) or IMT placebo group (IMTPG; *n* = 6). For IMT, resistance load was set at 50% and 15% of the maximum dynamic inspiratory strength (S-Index), respectively. Only the IMTG’s weekly load was increased by 5%. In addition, both groups carried out the same concurrent training. Besides the S-Index, a 1.5-mile running test, spirometry, and deoxyhemoglobin (HHb_AUC_ during occlusion) and reperfusion tissue saturation index (TSI_MB_ and TSI_MP_: time from minimum to baseline and to peak, respectively) in a vascular occlusion test were measured before and after the 4-week training program. In addition, resting heart rate and blood pressure were registered. Results: IMTG improved compared to IMTPG in the S-Index (Δ = 28.23 ± 26.6 cmH_2_O), maximal inspiratory flow (MIF: Δ = 0.91 ± 0.6 L/s), maximum oxygen uptake (Δ = 4.48 ± 1.1 mL/kg/min), 1.5-mile run time (Δ = −0.81 ± 0.2 s), TSI_MB_ (Δ = −3.38 ± 3.1 s) and TSI_MP_ (Δ = −5.88 ± 3.7 s) with *p* < 0.05. ΔVO_2max_ correlated with S-Index (r = 0.619) and MIF (r = 0.583) with *p* < 0.05. Both ΔTSI_MB_ and TSI_MP_ correlated with ΔHHb_AUC_ (r = 0.516 and 0.596, respectively) and with Δ1.5-mile run time (r = 0.669 and 0.686, respectively) with *p* < 0.05. Conclusion: IMT improves vascular function, which is related to additional improvements in physical performance.

## 1. Introduction

The respiratory adaptations recognized by inspiratory muscle training (IMT) include a decrease in the work of breathing, increased ventilatory efficiency, decreased perception of effort, lower signs of fatigue per unit of work, and attenuation of the metabolic reflex [1]. One of the main effects caused by these adaptations is an increase in physical performance. It should be noted that greater physical performance is related to better health condition and control of cardiometabolic risk factors in healthy subjects [2]. Therefore, the IMT can be used to improve health-related fitness.

Recently, additional direct effects on the cardiovascular system by IMT has been documented, particularly an improvement in autonomic modulation. This is characterized by an increase in vagal activity and a decrease in sympathetic activity [3]. As a consequence of this modulation, it is possible to observe systemic cardiovascular effects such as decreases in heart rate, peripheral vascular resistance, and blood pressure, among others [4]. In muscles, the decrease in sympathetic stimulation has been associated with improved vascular function related to higher vasodilatation. Previously, researchers have observed benefits in vascular function, attributed to decreased peripheral vascular resistance and increased nitric oxide bioavailability, following IMT [4,5]. In this way, a greater increase in blood flow to muscles during physical exercise can be expected [6], which will enable the maintenance of adequate oxygen delivery to the muscles according to the metabolic demands imposed, improving physical performance.

Using near-field infrared spectroscopy (NIRS), a portable and non-invasive technology, researchers have been able to evaluate the adaptations of vascular function and muscle metabolism [7,8]. For that, NIRS is used to measure the concentrations of total hemoglobin and its different physiological forms (i.e., oxyhemoglobin [O_2_Hb], deoxyhemoglobin [HHb]), as well as the tissue saturation index (TSI). In this sense, the most commonly used method is the analysis of changes in muscle [HHb] and [TSI] levels during ischemia and reperfusion periods in a vascular occlusion test (VOT) applied on a limb [9,10]. To date, this methodology has not been used to study the effect of IMT on muscle metabolism and vascular function. 

Therefore, understanding these phenomena could provide scientific support for the use and prescription of IMT for purposes of optimizing muscle adaptations to improve health-oriented physical fitness. The objective of this study was to determine the effects of an IMT program on metabolic and muscle vascular changes, and their relationship to changes in autonomic balance and physical performance in trained healthy subjects. 

## 2. Materials and Method

### 2.1. Sample Size

An a priori calculation was performed using the average change in peak dynamic inspiratory force (S-index: 34.3 ± 3.0 cmH_2_O) after IMT in physically active subjects. An alpha error of 5%, a statistical power of 80%, and an intergroup ratio of 1:1 was considered. A minimum of 6 subjects per group was estimated. Considering a loss-to-follow-up ratio of 25%, at least 8 subjects per group were considered for recruitment. Calculations were carried out with the G*Power version 3.1.9.7 software program (Düsseldorf, Germany). 

### 2.2. Subjects

Twenty healthy physically active male (age: 26.7 ± 2.2 years, weight: 75.3 ± 5.8 kg, height: 173.9 ± 5.5 cm, BMI: 24.9 ± 1.6 kg/m^2^) with at least 3 months of concurrent exercise training (CT) beforehand, which were randomized to an experimental group with IMT plus CT (IMTG; *n* = 10) or a placebo group with sham IMT plus CT (IMTPG; *n* = 10) for 4 weeks. For this purpose, an electronic randomization was performed (www.randomization.com). Normal-weight subjects were included who: (a) were ≥18 and ≤30 years old, (b) completed ≥95% of the total sessions, and (c) were physically active (≥150 min/wk). Those with previous IMT experience or who had an illness or injury that prevented them from performing or maintaining physical training were excluded. Prior to the initial assessments, subjects were required to sign a written informed consent form detailing the physical exercise assessments/interventions, benefits, potential complications, and management strategies to maintain the confidentiality of personal and measured data during the study. The evaluation and intervention protocols were approved by the Scientific Ethics Committee of the Universidad Viña del Mar (Code R62-19a). This research was conducted following the ethical considerations described in the Declaration of Helsinki for studies with humans.

### 2.3. Study Protocol

Subjects attended the first 3 sessions on different days for preintervention assessments. In the first session, anthropometric parameters, resting heart rate and blood pressure, and thigh skinfold measurements were obtained and familiarization of pulmonary function and vascular occlusion tests was performed. In the second session, spirometry, dynamic inspiratory force, and VOT tests were performed randomly. The third session was conducted on a running track where subjects performed a 1.5-mile run test. Then, after 4 weeks of CT and IMT (experimental or placebo), the same evaluations and in the same previous order were again performed. The sessions of both types of training were supervised by an exercise science professional. Only data from those subjects who met the inclusion criteria during the training period were analyzed (per protocol analysis).

### 2.4. Measurements

#### 2.4.1. Anthropometry and Autonomic Cardiac Parameters

Body weight was measured with a scale (803^®^, SECA, Hamburg, Germany), height was measured with a portable stadiometer (SECA, Hamburg, Germany). After 3 min of rest in a seated position, heart rate measured with a heart monitor (M400^®^, Polar, Kempele, Finland) and blood pressure measured with an automatic sphygmomanometer (HEM7156t^®^, Omron, Kyoto, Japan) were recorded to document the autonomic assessment.

#### 2.4.2. Spirometry and Maximal Dynamic Inspiratory Strength

Respiratory volumes and flows were measured with a portable spirometer (Microlab^®^, Viasys Healthcare, Warwick, UK). Before each evaluation, the equipment was calibrated with a 3-L syringe. Forced expiratory volume (FEV_1_), forced vital capacity (FVC), peak expiratory flow, and mean expiratory flow were obtained. The maximum voluntary ventilation was obtained by multiplying FEV_1_ × 37.5, and the Tiffenau index by dividing FEV_1_/FVC. The S-Index and peak inspiratory flow (MIF) were obtained with an electronic pressure meter (Kinetic-5^®^, POWERbreathe International Ltd., Southam, UK). The measurement was performed starting from the residual volume. Prior to the evaluation, a warm-up was carried out, as previously recommended. Both measurements were performed according to American Thoracic Society/European Respiratory Society (ATS/ERS) recommendations [11,12].

#### 2.4.3. Physical Performance

The time taken to perform a 1.5-mile running test on a 400 m Olympic track was measured with high-precision photocells (Wichro^®^, BoscoSystem, Spain). The estimated maximal oxygen uptake (VO_2max_) was determined as follows: VO_2max_= 65.404 + 7.707 × gender (male= 1; female= 0) − 0.159 × body mass (kg) − 0.843 × elapsed exercise time (min; walking, jogging or running), according to Larsen et al. [13]. The body weight used for the calculation was obtained with a scale prior to the exercise test (803^®^, SECA, Germany).

#### 2.4.4. Vascular Occlusion Test

A portable, wireless NIRS device (Portamon^®^, Artinis, Leiden, The Netherlands) was used to measure muscle oxygenation in real time during VOT. This dual wavelength system (760 and 850 nm) emits continuous-wave infrared beams from 3 LED emitters spaced 30, 35, and 40 mm from the receiver. The records are sent via Bluetooth to a computer program with a frequency of 10 Hz (Oxysoft, Artinis, The Netherlands). Using the Beer–Lambert law, changes in muscle oxygenation in terms of micromolar muscle concentrations of oxyhemoglobin [O_2_Hb], deoxyhemoglobin [HHb] and total hemoglobin [tHb] were measured. The tissue saturation index (TSI) is obtained as follows: %TSI = [O_2_Hb]/([O_2_Hb] + [HHb]) × 100. Before starting the test, the NIRS was placed on the vastus lateralis quadriceps 15 cm above the upper edge of the patella and 5 cm lateral to the midline of the thigh of the non-dominant leg in the direction of the muscle fibers. To maintain a reliable measurement, the NIRS was fixed with an elastic adhesive tape and covered with black cloth and an elastic bandage to avoid any disturbance by possible unintentional movement and/or interference from external light. The subjects were trained to remain still in the supine position during the measurements. Prior to NIRS installation, a skinfold of the area was measured with a caliper (Harpenden^®^, Holtain, United Kingdom) and blood pressure was taken. After 10 min of real-time reading stabilization, a pneumatic pressure cuff (within 5 s) installed around the proximal third of the thigh was rapidly inflated to 30 mmHg above the systolic pressure for 5 min. After this time, the cuff was rapidly deflated (within 1 s) and the NIRS recording was maintained for 5 min. The [HHb] and %TSI were used for the analyses. Both indexes were obtained from the sender at a distance of 40 mm, since this distance allows for greater penetration into the muscle [14]. Previously, [HHb] has been shown to be useful for measuring muscle oxidative metabolism; therefore, we used the calculation of the area under the deoxyhemoglobin curve (HHb_AUC_) to observe this condition [15]. To determine the baseline, [HHb] was analyzed during the last minute of the 10-min resting stabilization. The HHb_AUC_ was calculated just prior to the onset of occlusion until the end of the 5-min occlusion period, which was represented in arbitrary units. To calculate blood flow adaptations, the analysis of TSI during reperfusion had been previously validated [16]. We then obtained the time required for TSI to reach baseline (ΔTSI_MB_), i.e., up to the value obtained as baseline during rest, and the maximum value (ΔTSI_MP_) obtained during reperfusion at the time of cuff release (above the resting level), both from the minimum value reached during occlusion.

### 2.5. Intervention

#### 2.5.1. Concurrent Training

Concurrent training was conducted in five 80-min sessions per week (one per day) following the American College of Sports Medicine recommendations for improving fitness for health [17]. Each session included a 10-min warm-up with joint mobility, muscle activation and flexibilization exercises at low–moderate intensity, 30 min of moderate–vigorous aerobic exercise on a cyclo-ergometer or treadmill (65–70% of reserve heart rate), 30 min of strength exercises (70–80% of 1 maximum resistance) with 3 sets of 10 repetitions per muscle group with 2 min of rest per set. For the strength training sessions, the muscle groups were distributed as follows: Monday and Thursday: pectorals (pec deck fly or bench press), biceps (biceps curls), and deltoids (shoulder press or military press); Tuesday: triceps (pull over or skull crusher), forearm flexors and extensors (standing wrist curls and extensions), and latissimus dorsi (bent-over row or Kroc row). Wednesday: hip flexors and extensors (standing hip flexion and extension or hip thrust) and knees (hamstring curls and knee extension); Friday: latissimus dorsi, triceps, hip flexors and extensors, and knees; and Saturday and Sunday: rest. Finally, a 10-min cool-down with mobility, stretching, and relaxation breathing exercises was performed.

#### 2.5.2. Inspiratory Muscle Training

This consisted of 56 sessions distributed over 4 weeks with a Powerbreathe Plus medium resistance threshold device (Powerbreathe^®^, International Ltd., London, UK). Each day, 2 sessions were performed (1 am and 1 pm), each of 30 breaths at maximum intensity with a 50% S-Index load for IMTG, and 15% for IMTPG. Only IMTG increased by 5% per week. Prior to the start of each session, both groups performed a warm-up of 30 maximal inhalations at 15% of the S-Index. The sessions were monitored by a sports science professional. For subjects who had symptoms or any sign of discomfort caused by the exercise (e.g., headache, muscle pain, shortness of breath, etc.), the training would be suspended.

### 2.6. Statistic

Data are shown as averages and standard deviation. A Shapiro–Wilk test showed that the variables had a normal distribution. To compare changes between baseline and after intervention (∆) between groups in lung function, cardiorespiratory function, and muscle oxygenation, a two-way analysis of variance (two-way ANOVA) for repeated measurements and a Bonferroni post-hoc test was used. A Pearson correlation test was used to observe associations between the changes of the variables for all subjects. The interpretation of the levels of correlation coefficients was conducted according to Hinkle et al. [18], as follows: (i) 0 to 0.30 (0 to −0.30) was a negligible correlation; (ii) 0.30 to 0.50 (−0.30 to −0.50) was a low positive (negative) correlation; (iii) 0.50 to 0.70 (−0.50 to −0.70) was a moderate positive (negative) correlation; (iv) 0.70 to 0.90 (−0.70 to −0.90) was a high positive (negative) correlation; or v) 0.90 to 1.00 (−0.90 to −1.00) was a very high positive (negative) correlation. The statistical error considered for all significances was *p* < 0.05. All analyses were performed with Jamovi^®^ version 2.3.16 software [19]. The correlation figures were created in GraphPad Prism version 8.0.2 for Windows.

## 3. Results

Data recorded from eight (IMTG: 27.7 ± 2.2 years) and six subjects (IMTPG: 25.8 ± 2.6 years) who participated in 100% of the sessions were analyzed. Two subjects withdrew from the IMTG for not completing at least 90% of the training sessions. In IMTPG, two subjects were unable to continue with CT due to lower extremity musculoskeletal injury and two others did not attend post-intervention evaluations. Table 1 shows the baseline characteristics of both groups. Here, the BMI was higher in the IMTPG group with respect to the IMTG group with *p* = 0.038. This group presented an average BMI in the overweight range. 

Table 2 shows a significant decrease in HR_rest_ only in IMTG (Δ: −2.6 ± 2.9 bpm) with no difference with IMTPG (Δ: 1.0 ± 2.8 bpm). In addition, we observe an increase in cardiorespiratory and functional capacity for IMTG with significant difference with respect to IMTPG, represented by an increase in estimated VO_2max_ (Δ: 1.51 ± 2.5 versus 4.48 ± 1.1 mL/kg/min), and a decrease in 1.5-mile test time (Δ: −0.27 ± 0.4 versus −0.81 ± 0.2 s) with *p* < 0.05. A greater increase occurred in IMTG relative to IMTPG for S-Index (Δ: 28.23 ± 26.6 versus −13.83 ± 4.0 cmH2O) and FIM (Δ: 0.91 ± 0.6 versus −0.60 ± 0.1 l/s) with *p* < 0.05.

In Table 3, significant decreases in TSI_MB_ and TSI_MP_ for IMTG (Δ: −3.38 ± 3.1 and 0.83 ± 2.3 s) with respect to IMTPG (Δ: −5.88 ± 3.7 and 3.50 ± 6.4 s) were observed with *p* < 0.05. Likewise, HHb_AUC_ had a significant decrease between baseline and after intervention only in IMTG (Δ: −1336.1 ± 1462.5 a.u.) with *p* < 0.05. However, it did not differ with decreasing IMTPG; therefore, only one trend was observed (Δ: IMTG: −1336.1 ± 1462.5 a.u. versus −32.3 ± 259.3 a.u.; *p* = 0.054) (Table 3). 

**Figure 1:**
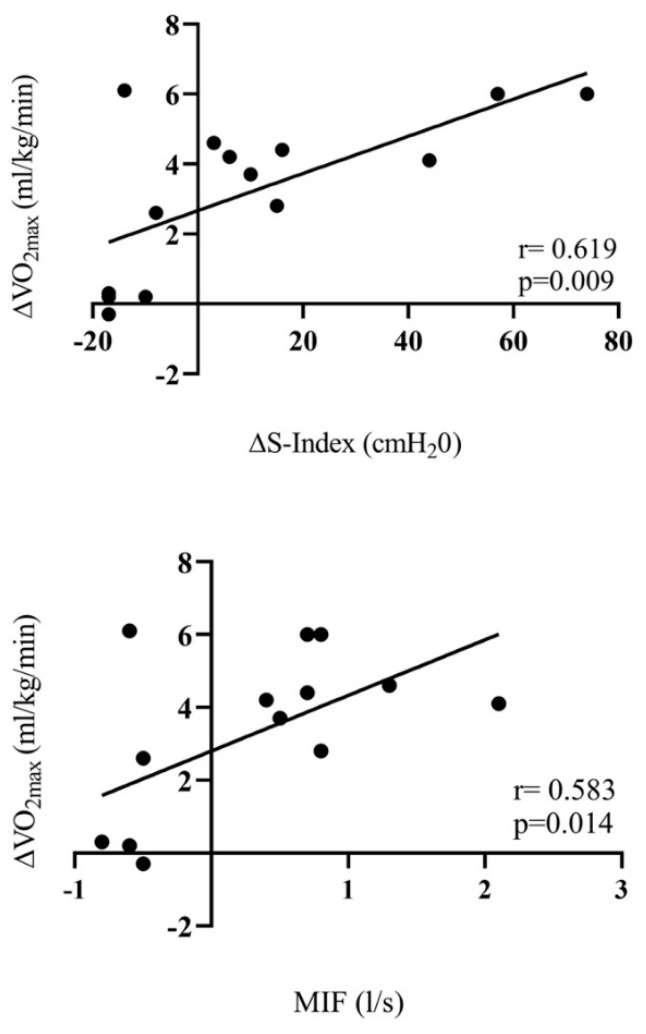
Correlation between ΔVO_2max_, ΔS-Index and ΔMIF.

Regarding the association of variables among subjects, the ΔS-Index (r = 0.619; *p* = 0.009) and MIF (r = 0.583; *p* = 0.014) had a moderate positive correlation with ΔVO_2max_ (Figure 1). 

**Figure 2:**
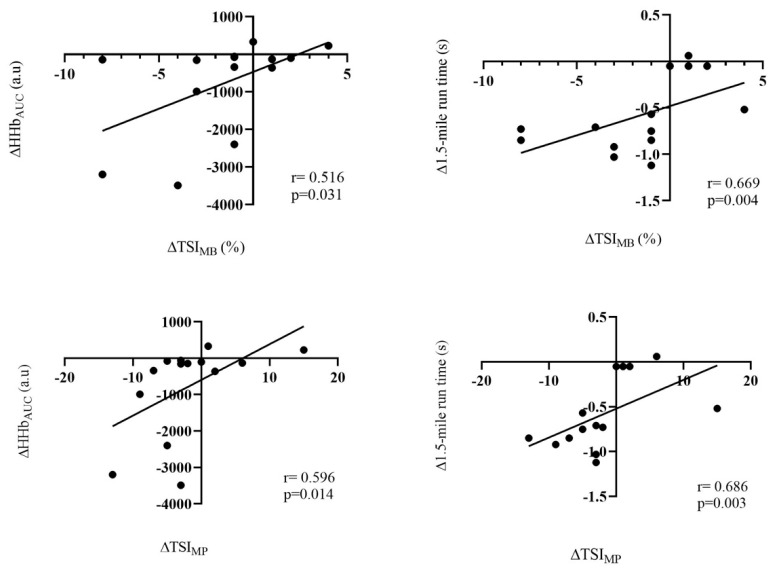
Correlation between ΔHHb_AUC,_ Δ1.5-mile run time, ΔTSI_MB_ and ΔTSI_MP_.

The ΔTSI_MB_ had a moderate positive correlation with ΔHHb_AUC_ (r = 0.516; *p* = 0.031), and with Δ1.5-mile run time (r = 0.669; *p* = 0.004). On the contrary, ΔTSI_MP_ had moderate positive correlations with ΔHHb_AUC_ (r = 0.596; *p* = 0.014) and with Δ1.5-mile run time (r = 0.686; *p* = 0.003) (Figure 2).

## 4. Discussion

The main results of this study showed that four weeks of IMT improved the peak inspiratory dynamic strength, maximum inspiratory flow, and quadriceps vascular function in healthy trained adults, favoring the increase of CRF and physical performance. Likewise, a trend in the increase of muscular oxidative capacity could be observed. However, we could not confirm an association between oxidative capacity and cardiovascular autonomic balance changes. 

A recent meta-analysis that analyzed clinical trials using IMT in subjects with or without health conditions (cardiovascular, *n* = 6; chronic kidney disease, *n* = 1; obstructive sleep apnea, *n* = 1; and healthy older adults, *n* = 2), demonstrated that this type of training can modulate the cardiovascular system, decreasing HR_rest_ and DBP. Here, the decrease in HR_rest_ was related to low (30% of the maximal inspiratory pressure) and moderate–high (50–70% of the maximal inspiratory pressure) resistance intensities for IMT, respectively [20]. In contrast to this, we observed a decrease in HR_rest_ in healthy adults trained at moderate intensity (50% of the S-Index), but with no changes in DBP. One of the explanations related to this cardiovascular response would be the execution of a greater number of repetitions [21]. Since most of these studies were carried out in people with some type of health condition (e.g., cardiac diseases), so that a higher number of repetitions has been used together with a lower intensity in order to avoid possible adverse effects of a higher inspiratory effort. However, this does not occur in healthy subjects, especially physically active ones, who have a greater capacity for post-training adaptive response. In this sense, we observed greater cardiovascular autonomic control for HR_rest_ in those who performed moderate-intensity (IMTG) exercise compared to those who performed low-intensity (IMTPG) exercise. It should be noted that both groups performed the same number of daily repetitions (*n* = 120), however IMTG performed 2 × 30 repetitions at low-intensity warm-up (15% of S-Index) plus 2 × 30 repetitions at moderate–high intensity (50% of S-Index), while IMTPG performed only low intensity during 5 days per weeks for 4 weeks. Therefore, it is possible to speak of short-duration strength endurance training in IMTG, and of moderate-duration strength endurance training in IMTPG. Now, with respect to DBP, it is possible that a longer intervention time may be necessary, as has been observed in other studies where changes were observed at 8 weeks [20]. 

A decreased sympathetic activity could favor vascular function (increased vasodilatation) in active muscle, especially during intense effort. Precisely, only in the IMTG group was there an increase in vascular function of the muscle and physical performance. However, these were not related to a greater decrease in autonomic modulation. It is possible that more sensitive mechanisms for recording autonomic activity may be necessary, such as heart rate variability, which can individually detect the vagal and autonomic sympathetic activities on the cardiovascular system [22]. On the contrary, bradycardia at baseline (HR_rest_ < 60 bpm) caused by high fitness levels in subjects may have limited further autonomic changes that could show differences between groups and the probability of a relationship with changes in physical performance.

An important point related to the increase in vascular function induced by the decrease in sympathetic activity in subjects exercising to improve health-related physical performance is the optimization of oxygen distribution to the active muscles [23]. Although a trend of improved muscle oxidative metabolism was observed only in the IMTG group, a trend was associated with changes in vascular function in the IMTG. Also, it should be noted that the increased strength of the inspiratory musculature will cause an inhibition of the metabolic reflex during exertion, preventing a sympathetic efferent vasoconstrictor effect on vascular function in the locomotor musculature, which is associated with increased physical performance [24,25,26]. In this sense, changes in inspiratory strength and vascular function were related to changes in CRF (VO2_max_) and physical performance (1.5-mile running), respectively. Precisely, these are the strengths of our study, which would provide evidence that a 4-week IMT improves physical performance of healthy subjects from changes related to respiratory and vascular function. The results of this study demonstrate that NIRS can be used by sport and health science professionals (e.g., clinical exercise physiologists, physical therapists, and others) to guide IMT programs aimed at improving muscle vascular function for health-related physical performance enhancement in healthy trained subjects.

A limitation of this study was the lack of measurement of gas exchange and ventilatory efficiency and muscle oxygenation during a cardiopulmonary exercise test (ergospirometry), which could have provided information on the likely inhibitory effect of the IMT on the metabolic reflex. Finally, due to the low final number of subjects per group, we do not recommend generalizing the results. 

## 5. Conclusions

In conclusion, an increase in dynamic muscle strength of the inspiratory musculature induced by a 4-week IMT program improved muscle vascular function in trained healthy adult subjects, which was related an increase in CRF and physical running performance.

## Figures and Tables

**Table 1:** Baseline characteristics of the study groups.

Variables	IMTPG (*n* = 6)	IMTG (*n* = 8)	Student’s *t*-Test
	Mean ± SD	Mean ± SD	
Age (years)	25.8 ± 2.6	27.7 ± 2.2	0.163
Weight (kg)	75.6 ± 6.7	74.3 ± 5.9	0.707
Height (m)	1.71 ± 0.1	1.76 ± 0.1	0.102
BMI (kg/m^2^)	25.9 ± 1.3	24.0 ± 1.6 **	0.038
ATT (mm)	10.5 ± 1.9	9.1 ± 2.6	0.300

ATT: Adipose tissue thickness, SD: standard deviation. Significant differences between (**) groups with *p*-value < 0.05.

**Table 2:** Lung function changes after inspiratory muscle training or placebo in trained subjects with concurrent exercise.

Variables	IMTPG (*n* = 6)			IMTG (*n* = 8)			ANOVA Test
	Baseline	After	Δ	Baseline	After	Δ	
	Mean ± SD	Mean ± SD		Mean ± SD	Mean ± SD		*p*-Value
*Cardiorespiratory fitness*							
HR_rest_ (bpm)	51.7 ± 5.2	52.7 ± 3.9	1.0 ± 2.8	54.4 ± 7.3	51.8 ± 5.0	−2.6 ± 2.9 **	0.037
SAP_rest_ (mmHg)	128.3 ± 9.6	128.8 ± 5.9	0.5 ± 6.2	124.3 ± 11.7	119.9 ± 8.6	−4.4 ± 5.4	0.141
DAP_rest_ (mmHg)	79.3 ± 7.1	77.0 ± 5.8	−2.3 ± 3.5	69.9 ± 5.8	67.5 ± 4.9	−2.8 ± 4.9	0.986
1.5-mile run(min)	9.85 ± 0.4	9.57 ± 0.6	−0.27 ± 0.4	9.84 ± 1.0	9.03 ± 0.9 *	−0.81 ± 0.2 **	0.006
VO_2max_ (mL/kg/min)	52.6 ± 1.7	54.1 ± 3.3	1.51 ± 2.5	53.1 ± 5.2	57.5 ± 6.0 *	4.48 ± 1.1 **	0.01
*Maximal dynamic inspiratory strength*							
S-index (cmH_2_O)	150 ± 6.5	136 ± 6.7	−13.83 ± 4.0	128 ± 22.9	156 ± 27.2 *	28.23 ± 26.6 **	0.003
MIF (L/s)	8.13 ± 0.3	7.53 ± 0.3 *	−0.60 ± 0.1	7.41 ± 1.2	8.32 ± 1.3 *	0.91 ± 0.6 **	<0.001
*Spirometry*							
FVC (L)	5.27 ± 0.5	5.15 ± 0.5	−0.11 ± 0.1	5.20 ± 0.6	5.27 ± 0.5	0.07 ± 0.2	0.08
FEV_1_ (L)	4.42 ± 0.4	4.28 ± 0.5	−0.15 ± 0.2	4.29 ± 0.6	4.44 ± 0.5	0.16 ± 0.5	0.214
FEV_1_/FVC_1_ (%)	83.8 ± 2.8	82.8 ± 2.2	−1.00 ± 1.6	82.6 ± 7.6	84.1 ± 4.6	1.50 ± 8.7	0.506
PEF (L/min)	605 ± 84.2	583 ± 69.4	−21.83 ± 41.1	591 ± 53.3	618 ± 63.8	26.38 ± 26.3 **	0.023
FEF_25–75%_ (L/s)	4.52 ± 0.7	4.34 ± 0.7	−0.18 ± 0.4	4.67 ± 0.8	4.70 ± 1.0	0.02 ± 0.5	0.415
MVV (L/min)	166 ± 16.3	160 ± 16.9	−5.50 ± 5.2	161 ± 20.7	167 ± 18.3	5.75 ± 2.2	0.212

FEV_1_: Forced expiratory volume in first 1 s, FVC: forced vital capacity, FEV_1_/FVC: relationship between FEV_1_ and FVC (Tiffeneau index), FEF_25–75%_: forced expiratory flow between 25% and 75% of the maximal flow, IMTG: inspiratory muscle training group, IMTPG: IMT-placebo group, MIF: maximal inspiratory flow, MVV: maximal voluntary ventilation, PEF: peak expiratory flow, S-Index: dynamic inspiratory muscle strength, SD: standard deviation, VO_2max_: maximal oxygen uptake. Δ: variation post-baseline intervention. Significant differences within (*) or between (**) groups with *p*-value < 0.05.

**Table 3:** Muscle oxygenation, vascular function, and metabolism during and after inspiratory muscle training or placebo in trained subjects with concurrent exercise.

Variables	IMTPG (*n* = 6)			IMTG (*n* = 8)			ANOVA Test
	Baseline	After	Δ	Baseline	After	Δ	
	Mean ± SD	Mean ± SD		Mean ± SD	Mean ± SD		*p*
TSI_baseline_ (%)	70.9 ± 3.9	68.8 ± 1.9	−2.13 ± 3.7	65.7 ± 3.9	68.4 ± 3.6	2.65 ± 3.4	0.028
ΔTSI_MB_ (s)	9.37 ± 4.3	10.02 ± 3.4	0.83 ± 2.3	10.54 ± 2.9	7.18 ± 1.9 *	−3.38 ± 3.1 **	0.015
ΔTSI_MP_ (s)	19.6 ± 4.8	23.1 ± 4.0	3.50 ± 6.4	20.3 ± 4.9	14.4 ± 2.0 *	−5.88 ± 3.7 **	0.004
HHb_AUC_ (a.u.)	4872 ± 1502	4840 ± 1545	−32.3 ± 259.3	5183 ± 1597	3847 ± 783 *	−1336.1 ± 1462.5	0.054

HHb_AUC_: area under the curve of deoxyhemoglobin, IMTG: inspiratory muscle training group, IMTPG: IMT-placebo group, SD: standard deviation, TSI: tissue saturation index, TSI_baseline_: last minute of %TSI during rest, ΔTSI_MB_: time from minimum to baseline %TSI during reperfusion, ΔTSI_MP_: time from minimum to peak %TSI during reperfusion. Δ: variation post-baseline intervention. Significant differences within (*) or between (**) groups with *p*-value < 0.05.

## Data Availability

Data and background information on the study can be obtained from the corresponding author.
